# Neurofibrome de l'avant bras: à propos d'un cas

**DOI:** 10.11604/pamj.2014.18.5.3774

**Published:** 2014-05-01

**Authors:** Hda Fahim, Khadija Hasnaoui

**Affiliations:** 1 Service de Chirurgie Orthopédique et Traumatologique, Hôpital Mohamed V, Séfrou, Maroc

**Keywords:** Neurofibromes, taches café au lait, imagerie, chirurgie, anatomie pathologie, neurofibromas, café au lait spot, imagery, surgery, pathology

## Abstract

Les tumeurs des nerfs périphériques sont rares et mal connues. Le diagnostic en est rarement fait avant l'intervention. Le traitement chirurgical est difficile, et risque d'entrainer des dégâts nerveux irréversibles s'il est mal conduit. Nous rapportons un cas de neurofibrome de l'avant bras dont la symptomatologie est souvent discrète associé à des taches café au lait sur la peau. De ce fait, l'imagerie et l'examen anatomopathologique ont une place importante dans la prise en charge de ces tumeurs.

## Introduction

Rarement isolé, le plus souvent associé à une maladie de Recklinghausen, le neurofibrome peut se présenter sous formes soit d'une tumeur cutanée (molluscum fibrosum) qui est une tumeur nerveuse, soit d'un neurofibrome proprement dit. Les neurofibromes représentent entre 10 et 20% des tumeurs des nerfs périphériques. Ils surviennent sans prédominance de sexe chez des adultes entre 20 et 30 ans.

## Patient et observation

Nous rapportons le cas d'un patient de 26ans qui a présenté une tuméfaction de l'avant bras gauche (1/3 moyen de la face antero-externe) apparue 6 mois auparavant, de taille et de volume croissants.

**Figure 1:**
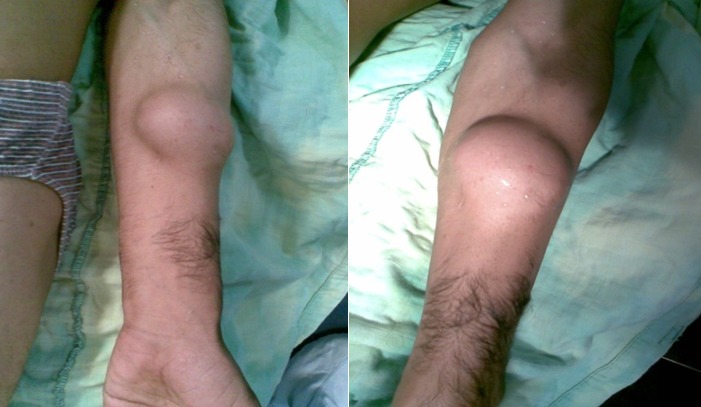
Vue de face et profil de la tumeur au niveau de l'avant bras

**Figure 2:**
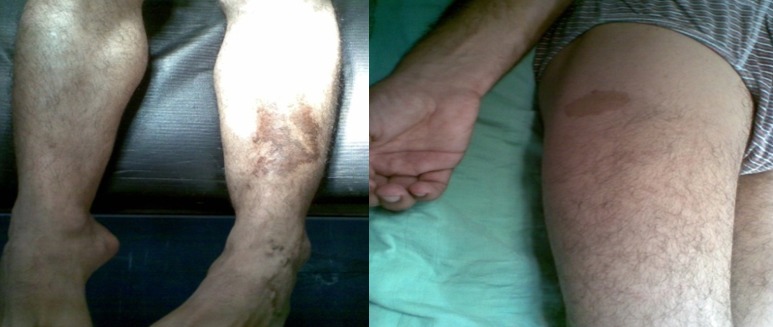
Taches café au lait au niveau de la cuisse et de la jambe

**Figure 3:**
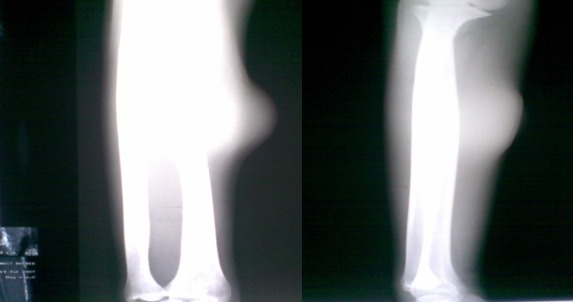
Opacité homogène des parties molles sans signes d'atteinte osseuse

**Figure 4:**
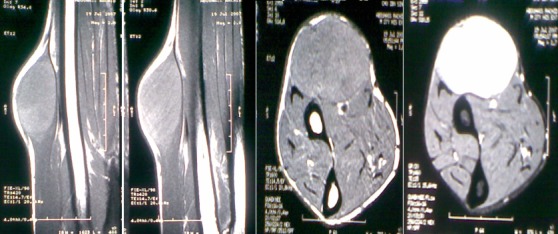
Aspect IRM (coronale et axiale) de la tumeur avant et après injection de produit de contraste

**Figure 5:**
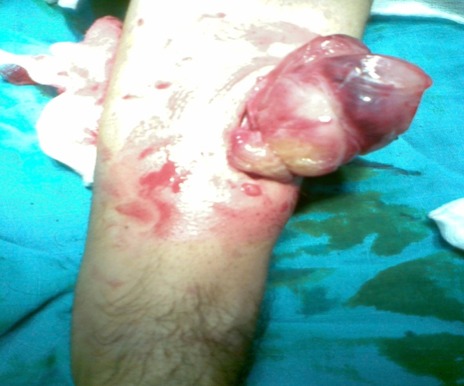
Vue per opératoire de la masse tumorale

**Figure 6:**
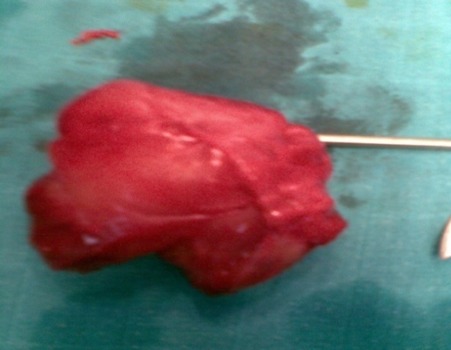
Aspect macroscopique de la tumeur

Il n'a rapporté aucune douleur, ni perte de poids, ni altération de l’état général, l'appétit est conservé, il a constaté uniquement l'apparition d'une gêne fonctionnelle croissante liée aux mouvements de l'avant bras, ainsi qu'une gêne sociale esthétique. Il n'a pas d'antécédents particuliers, notamment néoplasique. A l'examen clinique, on a observé une masse de la face antero-externe de l'avant bras mesurant 6/4 centimètres de grand axe. La lésion été mobile par rapport aux plans superficiels et profonds et légèrement dépressible à la palpation, d'apparence superficielle et les téguments sus-jacents été normo-colorés, sans télangiectasies, ni troubles vasculo-nerveux. Aucune Autre masse n'a été décelée ailleurs et les aires ganglionnaires sont libres (Figure 1). L'examen cutané a trouvé des Tâches café au lait localisé au niveau des membres supérieurs et inferieurs (cuisse, jambe, avant bras) (Figure 2). Le bilan biologique préopératoire a été normal. Les radiographies standards avant bras (face et profil) ont montré l'ombre de l'opacité de la tumeur sans lésions osseuses associées (Figure 3). L'IRM a montré une masse tissulaire de l'avant bras respectant les structures musculaires, vasculaires et l'os en regard. L'aspect en hyper signal T1 après Fat Sat fait évoquer une tumeur rare des parties molles (Figure 4:), la biopsie a été En faveur d'un neurofibrome. Une exérèse chirurgicale a été effectuée sous anesthésie générale (Figure 5). L'aspect macroscopique (Figure 6) a montré une tumeur englobant la branche terminale du nerf musculo-cutané (branches antérieure du nerf cutané latéral de l'avant-bras), cette tumeur a été difficilement dissociable et on a opté pour une résection en bloc de la tumeur alors que les deux extrémités du nerf ont été enfouillées en intramusculaires.

La tumeur ayant réalisé localement une légère expansion cutanée, une compression postopératoire et un drainage aspiratif été nécessaires pour éviter toute collection secondaire. Aussi bien la période peropératoire que postopératoire ont été sans particularité. Les résultats anatomopathologiques ont révélé une formation tumorale bénigne évoquant un neurofibrome. Apres un recul de 12 mois aucune récidive n'a été notée a part quelques troubles de sensibilité au niveau de l'avant bras.

## Discussion

Entre 60 et 90% des neurofibromes (toutes formes confondues), surviennent chez des patients indemnes de neurofibromatoses type 1 (NF1 ou maladie de Von Recklinghausen) [1].

Plusieurs formes de neurofibrome existent: - le neurofibrome localisé solitaire représente 90% de ces formes et dans la majorité des cas n'est pas associé à une NF1 [2, 3] - le neurofibrome plexiforme est pathognomonique de la NF1. Il survient fréquemment chez l'enfant et précède l'apparition des neurofibromes cutanés. Il correspond morphologiquement à un segment plus ou moins long de dilatation tortueuse d'un nerf et de ses branches, réalisant l'aspect en paquet de ficelle [4, 5]. Ces tumeurs sont de taille variable pouvant si on les laisse évoluer, devenir monstrueuses [6]. Classiquement situées prés des plis de flexion, ces tuméfactions peuvent siéger sur n'importe quel trajet nerveux.

Cliniquement la palpation doit être soigneuse, au niveau de la zone incriminée par le patient, mais aussi sur le trajet des principaux troncs nerveux. Elle peut permettre parfaitement de repérer la lésion, éventuellement d’évoquer une localisation précise sur un tronc nerveux, et de rechercher la présence d'un signe de Tinel lors de la percussion. Ce signe est noté dans 100% des tumeurs nerveuses périphériques (TNP) palpables. Une tuméfaction ovoïde palpable associée à un signe de Tinel est jusqu’à preuve du contraire une TNP [4, 7] La symptomatologie fonctionnelle est variable proportionnelle au volume de la tumeur. Souvent absente, ailleurs marquée par des douleurs et des signes distaux à type de paresthésies ou de signes déficitaires qui prennent souvent l'aspect d'une névralgie [1, 8].

L'examen général doit comporter l'inspection de tout le revêtement cutané pour mettre en évidence des lésions en faveur d'une NF de type 1 (taches café au lait, neurofibromes sous-cutanés, lentigines axillaires ou inguinales) et la recherche d'antécédents personnels, ou familiaux de Neurofibromatose [5]. A la palpation les neurofibromes sont fermes, hétérogènes, donnant parfois une sensation encéphaloïde, la percussion peut révéler un signe de Tinel, la peau en regard est souvent amincie.

L’échographie montre les rapports intimes entre la tumeur et le tronc nerveux. Elle est peu fiable dans le diagnostic de nature entre schwannome et neurofibrome, ou entre tumeur bénigne ou maligne. Le scanner apporte trois types de renseignements (la localisation au contact d'un tronc nerveux, l'aspect général de la lésion, la prise de contraste iodée est inconstante). L'imagerie en résonance magnétique, plus informative, est l'examen de choix pour le diagnostic des tumeurs des nerfs périphériques [4, 8–10]. Les neurofibromes sont classiquement considérés comme des tumeurs inextirpables. L'idéal serait de procéder à l'exérèse complète de la tumeur, sans dégât nerveux. Mais on sait que le neurofibrome est souvent adhérent aux tissus environnants, qu'il n'est pas encapsulé, et qu'il englobe souvent un certain nombre de fibres nerveuses [4, 11]. Certains auteurs ont proposé une résection en bloc de la tumeur et du tronc porteur, associée à une anastomose épi neurale ou une greffe fasciculaire dans le même temps [1, 4] d'autres ont préconisé l'utilisation du microscope opératoire [11, 12].

## Conclusion

Il est bien impératif d'enlever tout neurofibrome suspect de malignité, alors que les lésions bénignes ne doivent être opérées que s'ils sont gênants.
